# The Antinociceptive Effects of Rosuvastatin in Chronic Constriction Injury Model of Male Rats

**DOI:** 10.32598/bcn.9.4.251

**Published:** 2018-07-01

**Authors:** Amin Hasanvand, Fariba Ahmadizar, Abolfazl Abbaszadeh, Hossein Amini-Khoei, Mehdi Goudarzi, Amir Abbasnezhad, Razieh Choghakhori

**Affiliations:** 1. Department of Pharmacology and Toxicology, Faculty of Pharmacy, Lorestan University of Medical Sciences, Khorramabad, Iran.; 2. Department of Epidemiology, Erasmus University Medical Center, Rotterdam, Netherlands.; 3. Department of Surgery, School of Medicine, Lorestan University of Medical Sciences, Khorramabad, Iran.; 4. Medical Plants Research Center, Basic Health Sciences Institute, Shahrekord University of Medical Sciences, Shahrekord, Iran.; 5. Medicinal Plant Research Center, Ahvaz Jundishapur University of Medical Sciences, Ahvaz, Iran.; 6. Nutritional Health Research Center, Lorestan University of Medical Sciences, Khorramabad, Iran.; 7. Department of Nutrition, School of Health, Lorestan University of Medical Sciences, Khorramabad, Iran.

**Keywords:** Rosuvastatin, Neuralgia, Chronic Constriction Injury (CCI), Rats

## Abstract

**Introduction:**

According to studies, statins possess analgesics and anti-inflammatory properties. In the present study, we examined the antinociceptive, anti-inflammatory and antioxidative effects of rosuvastatin in an experimental model of Chronic Constriction Injury (CCI).

**Methods:**

Our study was conducted on four groups; sham, CCI (the control group), CCI+rosuvastatin (i.p. 5 mg/kg), and CCI+rosuvastatin (i.p. 10 mg/kg). We performed heat hyperalgesia, cold and mechanical allodynia tests on the 3^rd^, 7^th^, 14^th^, and 21^st^ after inducing CCI. Blood samples were collected to measure the serum levels of Tumor Necrosis Factor (TNF)-α, and Interleukin (IL)-6. Rats’ spinal cords were also examined to measure tissue concentration of Malondialdehyde (MDA), Superoxide Dismutase (SOD), and Glutathione Peroxidase (GPx) enzymes.

**Results:**

Our findings showed that CCI resulted in significant increase in heat hyperalgesia, cold and mechanical allodynia on the 7^th^, 14^th^ and 21^st^ day. Rosuvastatin use attenuated the CCI-induced hyperalgesia and allodynia. Rosuvastatin use also resulted in reduction of TNF-α, IL-6, and MDA levels. However, rosuvastatin therapy increased the concentration of SOD and GPx in the CCI+Ros (5 mg/kg) and the CCI+Ros (10 mg/kg) groups compared to the CCI group.

**Conclusion:**

Rosuvastatin attenuated the CCI-induced neuropathic pain and inflammation. Thus, antinociceptive effects of rosuvastatin might be channeled through inhibition of inflammatory biomarkers and antioxidant properties.

## Highlights

- Rosuvastatin administration significantly increases anti-inflammatory activity.
- Rosuvastatin administration significantly increases antioxidant activity.
- Rosuvastatin administration significantly increases antinociceptive activity.

## Plain Language Summary

Neuropathic pain includes the unpleasant sensations of burning and tingling, increased sensitivity towards the hyperalgesia and pain due to allodynia. Both hyperalgesia and allodynia coexist in the hyperinflammatory and neuropathic pains. Rosuvastatin, the cholesterol-lowering drug, activates the inhibitory reductase enzyme of 3-Hydroxy-3-Methylglutaryl-Coenzyme (HMG-CoA), which is widely used in the treatment of dyslipidemia and hypercalcemia. Recent studies suggest that rosuvastatin possesses dose-dependent antioxidant, anti-inflammatory, and analgesic activities. Several studies have shown that anti-inflammatory property of rosuvastatin through the leukocyte adhesion inhibition reduces the production of inflammatory mediators and their antioxidant effects.

The purpose of the current study was to assess the analgesic, anti-inflammatory and antioxidative effects of rosuvastatin in animals with neuropathic pain due to chronic constriction injury. Animals were randomly divided into four experimental groups (n=10 in each group): 1. Sham-operated; 2. CCI vehicle-treated (CCI); 3. CCI+Rosuvastatin (Ros) (5 mg/kg); and 4. CCI+Ros (10 mg/kg). The study results showed that the rosuvastatin was effective in reducing neuropathic pains, where the inflammatory factors play a key role. Rosuvastatin is an anticholesterol drug, which reduces the level of the inflammatory factors with its beneficial effects on neuropathic pain. Briefly, rosuvastatin can reduce the production of the inflammatory mediators and neuronal damage, thus improves the neuropathic pain in CCI model.

## 1. Introduction

Neuropathic pain (central or peripheral) is a complex chronic condition which results from nerve damage and dysfunction. Neuropathic pain symptoms include an unpleasant sensation of burning or tingling, increased sensitivity to the noxious stimuli (hyperalgesia) and pain due to innocuous stimuli (allodynia) (Dworkin et al., 2003). Central and peripheral mechanisms of Neuropathic Pain (NP) consist of changes in ion channel expression and nerve neurotransmitter release (Zhuo, 2007). Several factors are associated with neuropathic syndromes, including chronic disorders like diabetes and stroke, injury (spinal cord injury, multiple sclerosis), and infection (HIV-related neuropathies).

When tissues and nerves get injured, pain receptors are activated due to inflammatory stimulation (De Jongh et al., 2003). Inflammation is a potent modifier of responses to both noxious stimuli (hyperalgesia) and innocuous stimuli (allodynia) (Zanjani et al., 2006). Several experimental studies have shown that Reactive Oxygen Species (ROS) contribute to hyperalgesia and allodynia (Kim et al., 2004; Trevisan et al., 2016). Proinflammatory cytokines including Interleukin (IL)-6 and Tumor Necrosis Factor (TNF)-α play significant roles in neuronal reaction and inflammation. Both might also induce nerve injury.

Statins known as 3-Hydroxy-3-Methylglutaryl-Co-enzyme (HMG-CoA) reductase inhibitors are widely used as lipid-lowering medications to treat dyslipidemia (Murrow et al., 2012). HMG-CoA reductase is an enzyme of the mevalonate pathway that produces isoprenoids (with the exception of cholesterol synthesis). Studies have suggested that mevalonic acid has a significant role in intracellular events, e.g. apoptosis, inflammation, leukocyte migration, adhesion, and clotting (Coward, Marei, Yang, Vasa-Nicotera, & Chow, 2006). It has been shown that statins medications have anti-inflammatory, anti-oxidative, and neuronal protection effects in pathological conditions (Gholami et al., 2008; Koh, Sakuma, & Quon, 2011; Mohammadi, Amini, Jahanbakhsh, & Shekarforoush, 2013). Rosuvastatin, compared to the other statins, is a relatively potent HMG-CoA reductase inhibitor with a high degree of selectivity for liver cells (McTaggart, 2003).

The novelty of this research is to find out the analgesic, anti-inflammatory, and antioxidative effects of rosuvastatin in animals with neuropathic pain due to Chronic Constriction Injury (CCI).

## 2. Methods

### 2.1. Animals and housing conditions

Forty male adult Sprague–Dawley rats, weighing 200–250 g, were purchased from Razi Herbal Medicine Research Center (Khorramabad, Iran). Animals were housed at a temperature of 23±2°C, humidity of approximately 50%, and 12:12 h light/dark cycle with free access to water and standard food.

### 2.2. Study design

Animals were randomly divided into four experimental groups (n=10 in each group): 1. Sham-operated (Sh); 2. CCI vehicle-treated (CCI); 3. CCI+Rosuvastatin (Ros) (5 mg/kg) (Ferreira et al., 2014); and 4. CCI+Ros (10 mg/kg) (Mayanagi, Katakam, Gáspár, Domoki, & Busija, 2008). Rosuvastatin was injected once per day before the operation and continued daily until the 21^st^ day post-ligation.

### 2.3. Drug preparation

Rosuvastatin (Sigma-Aldrich, St. Louis, MO, USA) was suspended in distilled water as vehicle. Pentobarbital sodium (Sigma-Aldrich, St. Louis, MO, USA) was used for anesthesia. All drugs were prepared freshly and injected by the intraperitoneal (i.p.) route.

### 2.4. Operation

We used chronic constriction injury model to induce neuropathic pain in animals. The operation was performed under pentobarbital sodium (60 mg/kg) anesthesia. After anesthesia, skin and muscle were separated and left sciatic nerve was exposed. Next, we carefully tied 4 chromic gut ligatures loosely around sciatic nerves. The space between two adjacent ligatures was 1 mm. The wound was irrigated with sterile normal saline (0.9%) and sutured in two layers with non-absorbable sutures (fascial plane), and finally surgical skin staples. In the sham-operated group, the same surgical procedure (except the ligation) was performed (Bennett & Xie, 1988).

### 2.5. Thermal stimulation tests

In order to assess rosuvastatin effect on neuropathic pain, behavioral tests were recorded on days 3, 7, 14, and 21 after inducing CCI. The experiment started with mechanical test and terminated with cold allodynia. Sixty minutes were considered as the interval time of the two tests.

#### 2.5.1. Heat hyperalgesia stimulation (Hot Plate Test)

Heat hyperalgesia was measured using a hot plate test animal. Each animal was placed on the hot plate (temperature of 52.5±1.0°C). Afterward, paw withdrawal latency, with respect to licking of the hind paw and jumping, was recorded in seconds. The cut-off time of 10 seconds was maintained (Li et al., 2015).

#### 2.5.2. Cold allodynia (Acetone Test)

In this test, the animal’s hind paws were located over a wire mesh and acetone was sprayed onto its surface without touching the paw (100 μL). Then, the animal’s response to acetone was noted in 20 s and scored according to 4-point Kukkar and Singh scale. For obtaining a single score for a cumulative period of 60 s, individual scores for each 20 s intervals were added over. The score range was defined from 0 to 9 (Kukkar, Singh, & Jaggi, 2013).

#### 2.5.3. Mechanical allodynia (Von Frey Test)

Mechanical allodynia was determined by using von Frey filaments. When rats were adapted to the cages, the von Frey filament was applied by increasing the strength gently (2–60 g) of central region in plantar surface of hind paws until the animal lifts the paw away (Banafshe et al., 2012).

### 2.6. Elisa assay

In order to evaluate the serum levels of Tumor Necrosis Factor alpha (TNF-α) and Interleukin 6 (IL-6), on the day 21 after the operation, the blood sample was collected from the jugular vein. TNF-α and IL-6 levels were measured by solid phase sandwich ELISA kit specified for TNF-α protein and IL-6 (Cusabio, Biotech, Wuhan, Hubei, China). The analysis of TNF-α and IL-6 protein expression were calibrated according to the manufacturer’s instructions. Superoxide Dismutase (SOD), Glutathione Peroxidase (GPx) and Malondialdehyde (MDA) were estimated in fresh spinal cord after spinal dislocation. For this purpose, the rats’ spinal cords were isolated immediately on the day 21 after behavioral measurements.

Then, the homogenate tissue was prepared with 0.9% saline using glass homogenate and centrifuged at 2500 rpm for 10 min. Homogenate supernatant (10%, w/v) was used for these tests. Superoxide Dismutase (SOD) and GPx and Malondialdehyde (MDA) levels were estimated by using specific quantitative kits (Mohammadi et al., 2013).

### 2.7. Histological study

Sciatic nerves were separated on day 21 after operation. Histological studies were accomplished according to the protocol. Samples were then cut into 5- μM sections and stained with H&E. A pathologist who was blinded to the study analyzed the slides using a well-established scales for perineural measurement (Brummett, Padda, Amodeo, Welch, & Lydic, 2009).

### 2.8. Statistical analysis

Behavioral data were analyzed by 2-way ANOVA followed by a post hoc Tukey test. Also inflammatory cytokines, oxidative stress and pathological dates were analyzed by 1-way ANOVA followed by a post hoc Dunnett test. In all cases P≤0.05 was considered statistically significant. All data were expressed as mean±SED (Using GraphPad Prism Version 5.0).

## 3. Results

### 3.1. Rosuvastatin effect on heat hyperalgesia (Hot plate test)

**Figure 1.**
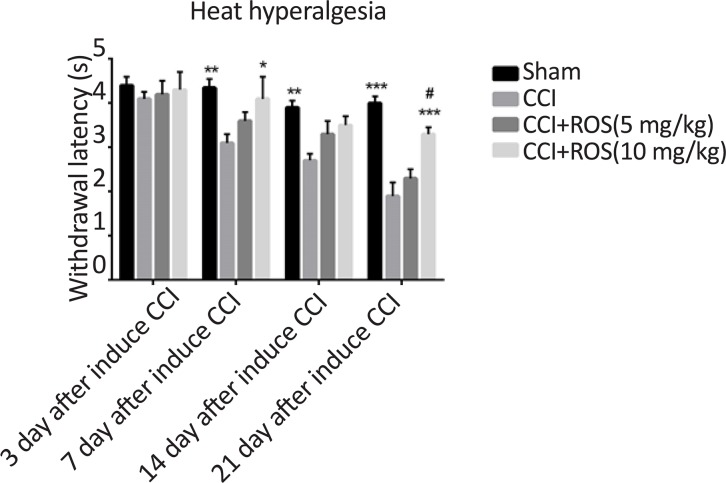
Effect of rosuvastatin treatment on hyperalgesia heat stimulation score in study groups Significant difference in heat stimulation score activity in the CCI+Ros (5 mg/kg) and CCI+Ros (10 mg/kg) groups compared to the sham group. Significant difference in heat stimulation score in the CCI+Ros (10 mg/kg) group compared to the CCI group. *P<0.05 vs CCI; **P<0.01 vs CCI; ***P<0.001 vs CCI; #P<0.05 CCI+Ros (5 mg/kg) vs CCI+Ros (10 mg/kg)

Figure 1 shows rosuvastatin effect on hyperalgesia heat stimulation in the treatment groups compared with the CCI group. The CCI model significantly showed increase in withdrawal latency time to hyperalgesia heat stimulation compared to the sham group on the 7^th^, 14^th^, and 21^st^ days of study (P<0.01, P<0.01 and P<0.001, respectively). In addition, there were significant differences between the CCI+Ros (10 mg/kg) and the CCI group on day 7 (P<0.05) regarding the heat stimulation score. Moreover, there was a significant difference between the sham and the CCI+Ros (10 mg/kg) group on the 21^st^ day of study (P<0.001). Also, a significant difference was observed between the CCI+Ros (5 mg/kg) and the CCI+Ros (10 mg/kg) group on day 21 (P<0.05). There was no significant difference between the CCI and the CCI+Ros (5 mg/kg) in all days.

### 3.2. Rosuvastatin effect on cold allodynia (acetone test)

**Figure 2.**
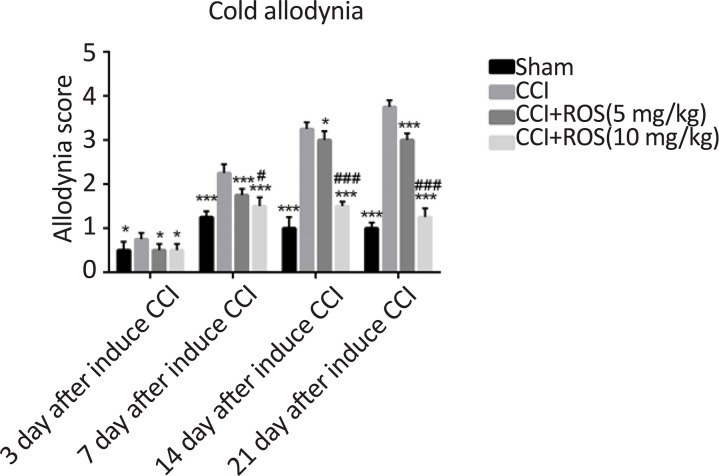
Effect of rosuvastatin treatment on cold allodynia stimulation test in study groups Significant difference was seen in heat stimulation score in the CCI+Ros (5 mg/kg) and CCI+Ros (10 mg/kg) groups compared to the CCI group. *P<0.05 vs CCI; **P<0.01 vs CCI; ***P<0.001 vs CCI; #P<0.05 CCI+Ros (5 mg/kg) vs CCI+Ros (10 mg/kg); ##P<0.01 CCI+Ros (5 mg/kg) vs CCI+Ros (10 mg/kg); ###P<0.001 CCI+Ros (5 mg/kg) vs CCI+Ros (10 mg/kg)

Figure 2 shows increasing response to the stimulation caused by acetone spray on the rats’ feet sole subject to the imperfect ligation of the sciatic nerve that indicates the induction of neuropathic pain among the CCI rats. These differences were between all groups compared to the CCI group on the 3^rd^, 7^th^, 14^th^, and 21^st^ days. In addition, there was significant differences in the CCI+Ros (5 mg/kg) compared to the CCI+Ros (10 mg/kg) group on the 7^th^, 14^th^ and 21^st^ days (P<0.05, P<0.001 and P<0.001, respectively).

### 3.3. Rosuvastatin effect on mechanical allodynia

**Figure 3.**
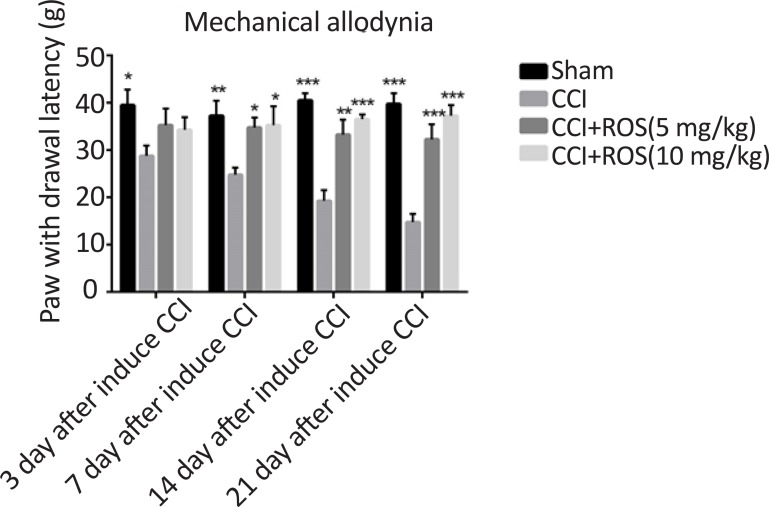
Effect of rosuvastatin treatment on mechanical allodynia stimulation test in study groups Significant difference was seen in heat stimulation score in the CCI+Ros (5 mg/kg) and CCI+Ros (10 mg/kg) groups compared to the CCI group. *P<0.05 vs CCI. **P<0.01 vs CCI. ***P<0.001 vs CCI. #P<0.05 CCI+Ros (5 mg/kg) vs CCI+Ros (10 mg/kg), ##P<0.01 CCI+Ros (5 mg/kg) vs CCI+Ros (10 mg/kg), ###P<0.001 CCI+Ros (5 mg/kg) vs CCI+Ros (10 mg/kg).

Figure 3 shows rosuvastatin effect on mechanical allodynia stimulation in the treatment groups compared with the sham and the CCI groups. The CCI model resulted in significant increase in paw withdrawal latency to mechanical allodynia stimulation compared to the sham group on the 3^rd^, 7^th^, 14^th^ and 21^st^ days of study (P<0.05, P<0.01, P<0.001, and P<0.001, respectively). In addition, there were significant differences in paw withdrawal latency in the CCI+Ros (5 mg/kg) (P<0.05, P<0.01 and P<0.001, respectively) and the CCI+Ros (10 mg/kg) (P<0.05, P<0.001, and P<0.001, respectively) compared to the CCI group on the 7^th^, 14^th^ and 21^st^ days. No significant differences were observed between the CCI+Ros (5 mg/kg) and the CCI+Ros (10 mg/kg) in all days.

### 3.4. Rosuvastatin effect on tumor necrosis factor-alpha and interlukin-6 protein analysis

**Figure 4.**
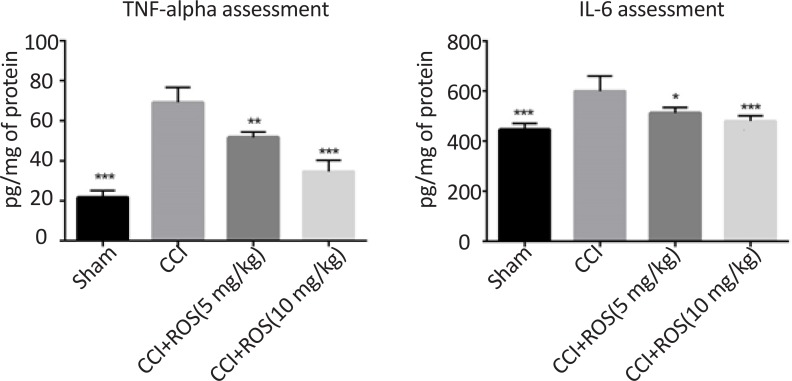
Effect of rosuvastatin treatment on TNF-α and IL_6 concentration in study groups Significant difference was observed in TNF-α and IL-6 activity in the sham, CCI+Ros (5 mg/kg) and CCI+Ros (10 mg/kg) groups compared to the CCI group. *P<0.5; ** P<0.01; and ***P<0.001 compared to the CCI group.

As shown in Figure 4, TNF-α and IL-6 concentration increased significantly compared to those in the sham group (P<0.001). Rosuvastatin use reduced the increment ratio of TNF-α and IL-6 concentration in the CCI+Ros (5 mg/kg) and the CCI+Ros (10 mg/kg) groups compared to the CCI group.

### 3.5. Rosuvastatin effect on malondialdehyde, super-oxide dismutase, and glutathione peroxidase

**Figure 5.**
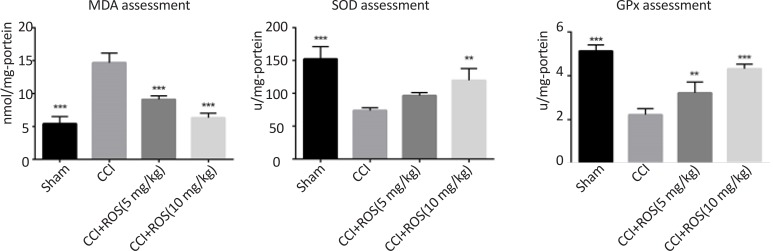
Effect of Rosuvastatin treatment on MDA, SOD and GPx activity in study groups Significant differences were seen in MDA, SOD and GPx activity in the sham, CCI+Ros (5 mg/kg) and CCI+Ros (10 mg/kg) groups compared to the CCI group. **P<0.01; and ***P<0.001 compared to the CCI group.

Figure 5 shows a significant increase in MDA activity in the CCI group compared to the sham group (P<0.001). The CCI+Ros (5 mg/kg) and the CCI+Ros (10 mg/kg) groups showed significant decrease in MDA activity as compared to the CCI group (P<0.001). Also, the result showed a significant decrease in SOD and GPx activities in the CCI group compared to the sham group (P<0.001). The CCI+Ros (10 mg/kg) showed a significant increase in SOD activity as compared to the CCI group, but not the CCI+Ros (5 mg/kg) group. As shown in Figure 5, both Ros (5 mg/kg and 10 mg/kg) administration significantly increased the GPx activity as compared to the CCI group (P<0.01 and P<0.001, respectively).

### 3.6. Rosuvastatin effect on histological study

**Figure 6.**
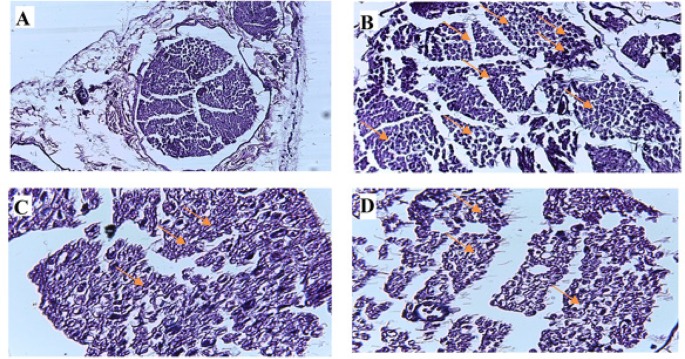
Histological and morphological studies in sciatic nerve at 21 days after CCI induction In an untreated CCI rats (B), the sciatic nerve has diffuse areas of moderate to marked edema/cellular infiltrate. In the Ros-treated (10 mg/kg) CCI rats (D), the finding is small focal areas of mild edema and/or cellular infiltrate. A: Sham; B: CCI; C: CCI+rosuvastatin (5 mg/kg); D: CCI+rosuvastatin (10 mg/kg)

Our result showed little signs of inflammation in the sciatic nerve in the sham group. Moreover, histological findings showed an extensive perineural inflammation in the sciatic nerve in the CCI. Also, the histological study showed a low inflammation ratio around the sciatic nerve in the CCI+Ros (10 mg/kg) group. In this study, nerve injury was not detected in sciatic nerve of all groups (Figure 6).

## 4. Discussion

CCI model is one of the most common models to induce neuropathic pain (Jaggi, Jain, & Singh, 2011). In our study, the CCI model significantly caused allodynia and hyperalgesia on the 21^st^ day after operation. We clearly demonstrated that rosuvastatin use (5 and 10 mg in a duration of 21 days) significantly improved behavioral changes of the induced CCI model, including heat hyperalgesia, cold allodynia and mechanical allodynia in the rat model of CCI-induced neuropathic nociception. Rosuvastatin treatment suppressed neuropathic-induced overexpression of inflammatory serum cytokines such as TNF-α, IL-6. Also, rosuvastatin attenuated the levels of oxidative markers like MDA, SOD and GPx in the spinal cord. The best result belonged to 10 mg/kg of rosuvastatin administration.

Rosuvastatin is categorized in the statin family, which can reduce the blood cholesterol levels through HMGCoA reductase pathway inhibition. Statins have independent cholesterol lowering effect, antioxidant effect (Uekawa et al., 2014), anti-inflammatory effect (de Vries et al., 2014), antibacterial effect (Lopez-Cortes et al., 2013), and most importantly neuroprotective effect (Wu et al., 2008). Many studies have shown various statins effects e.g. decreasing levels of inflammatory cytokines (Chu et al., 2012), restoring Neuronal nitric Oxide synthase (nNOS) expression (Ii et al., 2005) and antioxidant effect (Santos Fdo, Watanabe, Vasco, Fonseca, & Vattimo Mde, 2014); these effects might play important roles in pain improvement in neuropathic pain models. Proinflammatory cytokines such as IL-6, have been reported with their upregulating effect following nerve injury (Lee, Lee, Son, Hwang, & Cho, 2004). Inflammation activation is the main cause of chronic disease (Darabi, Hasanvand, & Nourollahi, 2016).

In our study, we found that neuropathic pain leads to increase in the level of inflammatory markers, including TNF-α and IL-6. It has already been demonstrated that TNF-α plays an important role in central and peripheral pains and enhances sensitivity to different stimuli (Leung & Cahill, 2010). However, treatment with rosuvastatin resulted in reducing TNF-α levels subjected to chronic constriction injury-induced by CCI. Several studies have shown that anti-inflammatory potential of rosuvastatin through leukocyte adhesion inhibition reduces the production of inflammatory mediators and its antioxidant effect. In another study conducted in 2006, rosuvastatin was shown to reduce intestinal ischemicreperfusion injury. Rosuvastatin was also associated with increase in serum nNOS level and to improve vascular structure (Naito et al., 2006).

Based on several studies, IL-6 may contribute to mechanical allodynia induced by spinal nerve lesion and CCI (Ramer, Murphy, Richardson, & Bisby, 1998; Murphy et al., 1999). Also, it has been found that SHR-CRP transgenic mice treated with rosuvastatin reduces the levels of the inflammatory factors, e.g. TNF-α and IL-6, and subsequently reduces the inflammation and oxidative damages (Silhavy et al., 2014). On the other hand, rosuvastatin reduces glial cells and lowers IL-1 levels and thus reduces the damages caused by neuropathic pain (Shi, Lim, Lee, Zhao, & Zhang, 2011).

Rosuvastatin plays a significant role in improving persistent pain and pain behaviors (Siniscalco et al., 2007). Preclinical studies have supported that the oxidative stress could enhance neuropathic pain and hyperalgesia which are strongly induced by peripheral nerve and or spinal cord injury in animal models of persistent pain (Kim, Wang, Lu, Chung, & Chung, 2009). Rosuvastatin use has a protective effect on oxidative stress by inducing Superoxide Dismutase 1 (SOD1) expression (Verreth et al., 2007). Enhancing the expression of glutathione synthase, GPx, glutathione reductase, and glutamylcysteine synthetase has been also related to rosuvastatin use (Mahalwar & Khanna, 2013). In a previous study, it has been shown that rosuvastatin possesses antioxidant, anti-inflammatory, and analgesic activities in a dose-dependent manner (Ghaisas, Dandawate, Zawar, Ahire, & Gandhi, 2010).

Rosuvastatin has beneficial effects in neuroprotective activity against spinal cord ischemia/reperfusion injury (Die, Wang, Fan, Jiang, & Shi, 2010), ischemic brain injury (Savoia, Sisalli, Di Renzo, Annunziato, & Scorziello, 2011), traumatic brain injury (Sanchez-Aguilar et al., 2013), and l-glutamate-induced excitotoxicity (Domoki et al., 2010). Finally, it has been shown that rosuvastatin has protective effects on nerve (Savoia et al., 2011; Yavuz et al., 2013).

In summary, rosuvastatin was effective in reducing neuropathic pains. Rosuvastatin use decreases inflammatory markers and oxidative stress. Our findings support the anti-inflammatory and antioxidant effects of rosuvastatin in the CCI-induced neuropathic pain in animal model. Also, our study suggests that rosuvastatin may have a neuroprotective effect in chronic constriction injury.

## Ethical Considerations

### Compliance with ethical guidelines

Animals were housed in temperature at 23±2°C, humidity of approximately 50% and 12-h light/dark cycle with free access to water and standard food. The ethics committee approved all of ethical guidelines of experimental pain in awaked animals. All tests were executed according to the guide for the care and use of laboratory animals (National Institutes of Health Newsletter No. 80-23, revised 1996).
